# A Novel, Reverse-Phase-Shifting, Thermoreversible Foaming Hydrogel Containing Antibiotics for the Treatment of Traumatic Tissue Injuries in a Swine Model

**DOI:** 10.1093/milmed/usaf085

**Published:** 2025-09-16

**Authors:** Ross I Donaldson, Aslam A Akhtar, Arghavan Farzadi, Todd L Graham, Jonathan K Armstrong, Oliver J Buchanan, John S Cambridge, Nely N Cristerna, Diane Goldenberg, Juliana Tolles, Maja Engler, James D Ross

**Affiliations:** Critical Innovations LLC, Los Angeles, CA 90260, USA; Department of Emergency Medicine, David Geffen School of Medicine at UCLA, Los Angeles, CA 90095, USA; Department of Emergency Medicine, Harbor-UCLA Medical Center, Torrance, CA 90509, USA; Department of Emergency Medicine, Harbor-UCLA Medical Center, Torrance, CA 90509, USA; Critical Innovations LLC, Los Angeles, CA 90260, USA; Benchmark Biotech LLC, Portland, OR 97232, USA; Critical Innovations LLC, Los Angeles, CA 90260, USA; Critical Innovations LLC, Los Angeles, CA 90260, USA; Critical Innovations LLC, Los Angeles, CA 90260, USA; Critical Innovations LLC, Los Angeles, CA 90260, USA; Critical Innovations LLC, Los Angeles, CA 90260, USA; Department of Emergency Medicine, David Geffen School of Medicine at UCLA, Los Angeles, CA 90095, USA; Department of Emergency Medicine, Harbor-UCLA Medical Center, Torrance, CA 90509, USA; Benchmark Biotech LLC, Portland, OR 97232, USA; Benchmark Biotech LLC, Portland, OR 97232, USA

## Abstract

**Introduction:**

More than one-third of patients with a combat injury evacuated to the United States have wound-related infections during their initial hospitalization, with prevention of such infections an ongoing battlefield challenge. Use of a reverse-phase-shifting thermoreversible foaming hydrogel containing vancomycin and tobramycin (HA) is one possible solution to this problem.

**Materials and Methods:**

This study compared HA to the hydrogel vehicle without antibiotics (HV) and control (i.e., no intervention). Bilateral, 5 cm diameter, full-thickness wounds were created in 12 male swine (∼50 kg, *Sus scrofa*). These wounds were then inoculated with *Staphylococcus aureus* and *Pseudomonas aeruginosa*. Ten minutes after inoculation, the wounds were treated with HA, HV, or no intervention. This treatment was reapplied daily for a total of 7 days. Blood samples were drawn at baseline, 1 h after initial administration, and on days 1, 3, and 7. After 7 days, the animals were humanely euthanized and the wounds were swabbed or biopsied for microbiological analysis and culture. Full-thickness tissue specimens were subsequently collected for blinded histopathological evaluation of wound healing and bacterial growth.

Institutional Animal Care and Use Committee and the U.S. Army’s Animal Care and Use Review Office approvals were received prior to initiation of animal experiments.

**Results:**

Bacterial growth and histopathology analyses of cutaneous and skeletal muscle biopsies at the end of the study showed less severe and/or less frequent intralesional bacterial and neutrophilic inflammation in the HA group compared to the HV and control groups. The HV and control groups were comparable, showing no significant differences in treatment effects. Statistical significance was observed in reduced bacterial infiltration when comparing the HA group with both the control and HV groups. Neither vancomycin nor tobramycin was detected systemically in any plasma sample at all time points, with a lower limit of quantification of 50 ng/mL for both antibiotics.

**Conclusion:**

Histopathology demonstrated significantly lower bacterial growth in the HA versus control (*P* = 1.4e^−4^) and HV (*P* = 3.2e^−4^) groups. It also showed statistically significant differences in other secondary outcomes for wound healing (e.g., neutrophilic infiltrate, fibroplasia, edema) between HA versus HV, as well as HA versus control, in both skin and muscle. In this porcine model of infected traumatic soft tissue injury, HA demonstrated significant benefits, and further research and development to explore its use in a range of settings is justified.

## INTRODUCTION

Recent advances in the care of traumatic injuries on the battlefield have led to improved survivability.^1–3^ However, this improvement has corresponded with a rise in the proportion of trauma-related infections that lead to severe ongoing morbidity.^3^ More than one-third of patients with a combat injury evacuated to the United States have wound-related infections during their initial hospitalization.^3^ Skin and soft-tissue infections and osteomyelitis are the most common wound infections in this population and frequently lead to increased long-term disability if not controlled early.^3^

Data from the Trauma Infectious Disease Outcomes Study, an in-depth study designed to better understand the disease burden and improve the outcomes of wounded patients, reveal that these wounds are frequently polymicrobial. The most commonly growing bacteria include *Enterococcus faecium, Pseudomonas aeruginosa, Acinetobacter spp*., and *Escherichia coli*.^4^  *Staphylococcus aureus* and *Staphylococcus epidermis* are also common and may present as delayed isolates. These bacteria make up a conglomerate known as the ESKAPE pathogens (***E****nterococcus faecium, **S**taphylococcus aureus, **K**lebsiella pneumoniae, **A**cinetobacter baumannii, **P**seudomonas aeruginosa, and **E**nterobacter spp*.), which are multidrug-resistant and represent a major therapeutic challenge.^5^ Preventing and decreasing morbidity related to such infections would lead to improved quality of life for wounded personnel, while also addressing a crucial need in infectious disease management.^5^

Due to the nature of combat, preventing battlefield wound infection is challenging and existing products have numerous limitations when applied to large combat wounds.^6^ Current triple-antibiotic (neomycin-bacitracin-polymyxin) ointments are contraindicated in large wounds due to the possible systemic absorption of their antibiotics which can result in toxicities.^7,8^ Existing hydrogels are also difficult to apply to deep, scattered, and larger surface area wounds. Gel products that are supplied in a tub with a spatula cannot feasibly be used in the field due to the cumbersome application process. Cut-out gels pads are also awkward to apply when wounds are large, scattered, or irregularly shaped.^9,10^ Additionally, the need for frequent removal and re-evaluation of the wound makes the above products largely impracticable for many clinical settings.

**Figure 1.**
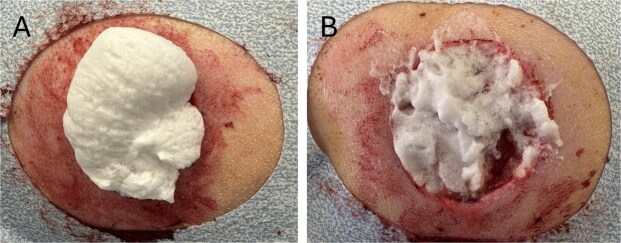
(A) Foam upon initial application. (B) Collapsed foam after ∼10 min of wound contact.

To address this need, Critical Innovations LLC has designed a reverse-phase-shifting thermoreversible foaming hydrogel containing broad spectrum antibiotics (HA) for the treatment of traumatic wounds in resource-limited settings.^11^ This product is supplied in a pressurized spray canister that can fit into an individual first aid kit. The product is sprayed directly onto the wound and can be deployed on a wide range of injury shapes. The foam conforms to the wound and strengthens to a viscous protective hydrogel within 10-15 min (Figure 1A and B). It is designed to function as a protective barrier to maintain a moist wound healing environment, while protecting from debris. Additionally, to provide antimicrobial protection for the commonly encountered pathogens discussed above, the hydrogel contains two antibiotics that together should result in broad-spectrum coverage: tobramycin (∼0.1%) and vancomycin (∼0.4%). The product is also designed to be removable if needed, through the application of room temperature water or saline, as the polymer solution’s reverse-phase-shifting thermoreversible polymers allow re-transition from a gel to a liquid based on temperature.

A recent pilot study by our group demonstrated that this product prevents bacterial colonization in a porcine model of thermal burns.^11^ Based on these findings, we hypothesized that this antibiotics-containing hydrogel would promote wound healing and prevent infection in a large-animal model of traumatic tissue injury.

## METHODS

### Regulatory Information

Approval was obtained from the Legacy Research Institute’s Institutional Animal Care and Use Committee and the U.S. Army’s Animal Care and Use Review Office (146-2023 and 21,000,280.e001, respectively). Experiments were conducted in a facility accredited by the Association for Assessment and Accreditation of Laboratory Animal Care at Legacy Research Institute, Portland, Oregon. Animals were used in accordance with The Guide for the Care and Use of Laboratory Animals.

### Materials

Canisters of HA and hydrogel vehicle without antibiotics (HV) were obtained from Critical Innovations LLC. Lyophilized *Staphylococcus aureus* (ATCC 27,853) and *Pseudomonas aeruginosa* (ATCC 12,600) were obtained from ATCC, Manassas, Virginia.

### Animal Selection and Sample Size Justification

Adult Yorkshire swine were chosen due to their wound healing characteristics paralleling those of humans.^12^ The specific traumatic injury model used was based on prior work developed at the U.S. Army Institute of Surgical Research,^13^ with adaptations in wound size and bacterial inoculation to mimic specific battlefield-wound environments. The number of animals used was determined *a priori* and was predicted to produce 80% power to detect an odds ratio of 0.1 associated with HA versus control (i.e., no intervention), assuming a type I error of 5%. Only animals that appeared healthy, robust, and free from significant disease or defect were enrolled. Twelve male swine (48.6 ± 1.4 kg, *Sus scrofa*) were used, each with contralateral wounds to serve as intra-animal comparisons.

### Wound Creation and Bacterial Inoculation

Animals were premedicated with 0.2 mg/kg meloxicam then sedated with 4-8 mg/kg Telazol® (Zoetis) and 0.04 mg/kg of atropine. They were induced via face mask with up to 5% isoflurane and 100% oxygen. They were then intubated, given 0.005-0.01 mg/kg of buprenorphine for analgesia, placed on a ventilator, and kept on 1-3% isoflurane to maintain a surgical plane. After hair removal and surgical scrub-preparation, bilateral 5 cm diameter full-thickness skin wounds were created over the gluteus maximus muscle using surgical incision for the perimeter of the wound and blunt dissection for the removal of the skin flap. Full thickness was defined as a complete removal of dermal tissues and subcutaneous fat to the level of the underlying muscle body; exact depth varied dependent on tissue layer thicknesses. The muscle tissue deep to the full-thickness skin wound was superficially injured using blunt dissection in a pattern of 1 cm increments across the entirety of the wound bed (5 total muscle wounds per wound bed). Bacterial inoculum of *S. aureus* and *P. aeruginosa* were prepared to a concentration of 3.80 × 10^7^-2.15 × 10^8^ colony forming units. The bacterial count was confirmed by serial dilution, plating, and counting. The bacteria solutions were pipetted topically onto the wound beds, separately and dispersed across its surface with a sterile instrument in a spiral fashion, from the center to the outside of the wound edge. Each wound bed on an animal received the same concentration and combination of bacterial inoculation. Across all animals, this resulted in the creation of a total of 24 wound beds (12 animals × 2 per animal).

### Application of Test Materials and Animal Recovery

Ten minutes after bacterial inoculation, each of the 24 wounds were randomized into one of three study groups (*n* = 8 per group). Each wound was treated with either HA, HV, or control. All wounds were then covered with Kerlix sterile gauze (Covidien) and covered with Tegaderm dressing (3 M) to minimize the chances of cross-contamination between interventions. After 1 h, the animals were weaned off anesthesia and returned to their housing. Each day after the initial injury procedure, dressings were removed from all wound sites and the wounds were photographed. For wounds randomized to HA or HV, the same article was re-applied, then covered with petroleum gauze (Curad^R^) along with the same dressing materials described above. Control wounds were only covered with dressing materials.

### Plasma Samples, Euthanasia, and Histopathology

K_2_EDTA plasma samples at baseline, 1 h, and 1-, 3-, and 7 days after treatment were analyzed for vancomycin and tobramycin concentration using liquid chromatography–tandem mass spectrometry (LC–MS/MS) method by a third-party laboratory (Alturas Analytics, Inc.). The LC–MS/MS system included a Shimadzu High Performance Liquid Chromatography system coupled to a LC-30AC pump, SIL-30AC autosampler and SCIEX API-6500 mass spectrometer. Compounds were chromatographically separated on a Synergi Hydro-RP column with gradient elution using water and acetonitrile, each containing 0.05% Heptafluorobutyric acid, as mobile phases A and B, respectively. The flow rate was 0.7 mL/min, and the total run time was 4 min and the assay range was set from 50 ng/ml to 5000 ng/ml antibiotic detection.

At the end of the 7-day observation period, all animals were humanely euthanized with 1 mL/10 lb of Euthanasia Solution (VetOne) while under general anesthesia. After dressing removal, each wound was swabbed using a culturette in a spiral fashion from the center to the outer portion of the wound edge. For histopathology, full thickness biopsies were also collected in 10% neutral buffered formalin, then embedded in paraffin, sectioned, mounted on slides, and stained with hematoxylin and eosin. These samples were analyzed by a third-party laboratory (IDEXX BioAnalytics) blinded to the study groups.

### Outcomes and Analysis

The primary outcome was bacterial colonization on histopathologic analysis at study end (day 7), quantified on 5-category ordinal scale (0-4). To objectively assess changes in the groups, microscopic changes were graded, as to severity, utilizing a standard grading system whereby 0 = no significant change, 1 = minimal, 2 = mild, 3 = moderate, and 4 = marked. The International Harmonization of Nomenclature and Diagnostic Criteria standards were used as the basis of evaluation.^14^ Use of numerical grades allowed a mechanism to calculate a total score lesion score, which could be used to assess prevalence and severity of tissue changes within and between groups.

The primary analysis was a cumulative logistic regression with a fixed effect for treatment group (control, HA, and HV) and a random effect to account for within-animal clustering. However, the prespecified analysis of a cumulative logistic regression model did not converge, likely representing the sparseness of the dataset; specifically, the consistent lack of bacterial presence in the wounds treated with HA and heavy growth in the control group. Thus, a post-hoc analysis was performed using two approaches. First, a Kruskal-Wallis test with a Bonferroni correction was conducted separately for each tissue type examined (muscle and skin), with the understanding that the outcomes from the two tissue types at the same wound site were correlated. Second, follow-up Wilcoxon tests for pairwise treatment comparisons were then performed. Given that clustering within animals was not accounted for by this strategy, an additional sensitivity analysis was conducted using a generalized estimating equation with a linear link function and a random effect for animals. This analysis was performed for skin and muscle samples independently.

The secondary outcomes of neutrophilic inflammation, macrophagic inflammation/foreign body response, ulceration, fibroplasia with neovascularization, dystrophic mineralization, and edema, each scored on a 5-category ordinal scale, were also analyzed separately with a cumulative logistic regression. An additional secondary outcome was bacterial counts from aerobic culture and ID tests.

## RESULTS

**Figure 2.**
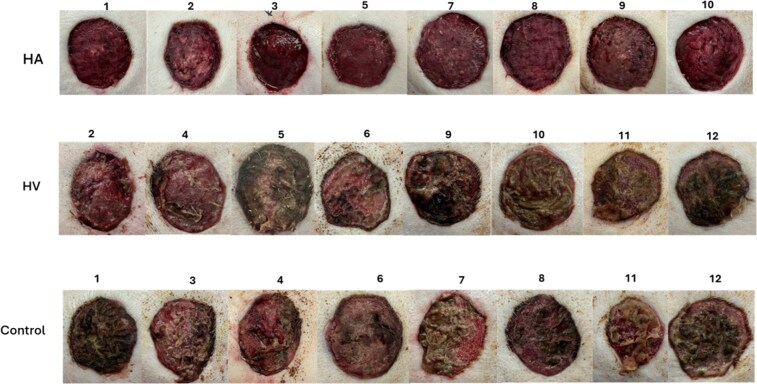
Pictures of the wounds at experiment end (Day 7): HA (top row), HV (middle row) and Control (bottom row) groups. HA, Hydrogel containing antibiotics; HV, Hydrogel vehicle without antibiotics.

All 12 swine (48.6 ± 1.4 kg) survived to the conclusion of the study (day 7), with a total of 24 wounds harvested. Pictures of the wounds on at experiment end (day 7) are shown in Figure 2.

The average masses of HV and HA applied topically daily were 2.9± 0.9 g. Average masses of vancomycin and tobramycin in applied HA were 9.2 ± 5.4 mg and 1.9 ± 1.1 mg, respectively. The blood samples drawn from baseline, 1 h, and 1-, 3-, and 7 days after treatment all demonstrated no detection of vancomycin or tobramycin (LLOQ of 50 ng/mL for both antibiotics).

**Figure 3.**
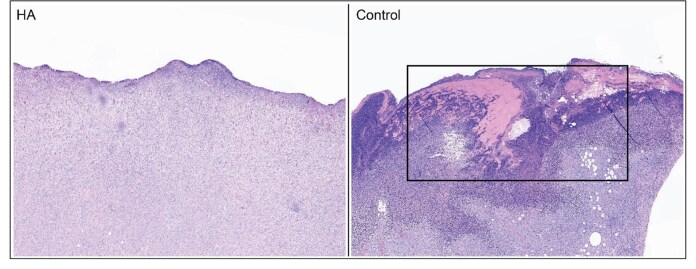
Side by side comparison of an example wound (center) on hematoxylin and eosin stain with 4× Magnification. Left (HA) versus Right (Control). The HA sample exhibits ulceration with mild suppurative inflammation and severe neovascularization and fibroplasia. The control sample exhibits severe suppurative inflammation, severe neovascularization, and fibroplasia. A large number of bacteria (rectangle) is identified in the control specimen, whereas the HA specimens did not contain a significant number of bacteria. HA, Hydrogel containing antibiotics.

**Figure 4.**
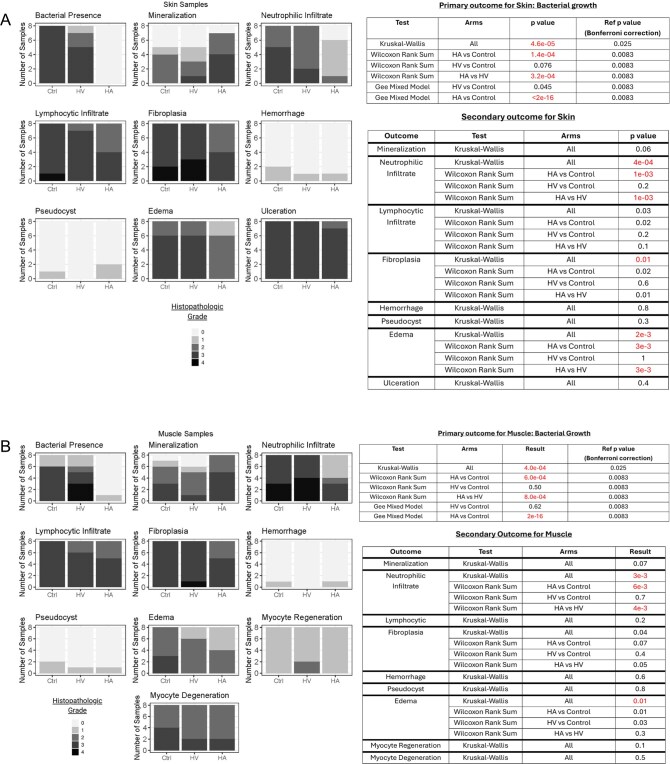
Results of histopathology statistical analysis for skin (A) and muscle (B) samples. Histopathologic grade: 0 = no significant change, 1 = minimal, 2 = mild, 3 = moderate, and 4 = marked.

Histopathology demonstrated significantly lower bacterial growth in the HA versus control (*P* = 1.4e^−4^) and HV (*P* = 3.2e^−4^) groups (Figures 3 and 4). Similarly, it also showed significantly lower neutrophilic infiltrates as compared to these 2 groups as well (*P* = .0014 and *P* = .001, respectively). Data also demonstrated statistically significant differences in other secondary outcomes for wound healing (e.g., fibroplasia, edema) between HA versus HV, as well as HA versus control, in both skin and muscle (Figure 4).

## DISCUSSION

To our knowledge, this is the first study to describe translational efficacy of a novel antibiotic-containing reverse-phase-shifting thermoreversible foaming hydrogel (HA) in live animal model with full thickness wounds at high infection risk. This model was adapted from previously developed traumatic tissue methodology^13^ with changes in wound size and the addition of inoculation with both gram-positive and gram-negative bacteria. Overall, this full-thickness model demonstrated consistent injuries across all animals and significant signs of infection in the control arm.

The primary outcome of this study was bacterial presence on histopathologic analysis using a validated ordinal scale at study end. Although the prespecified analysis did not converge (likely due to the sparseness of the dataset), post-hoc analysis demonstrated that HA treated wounds had statistically less bacterial presence when compared to both vehicle (HV) and control for both skin and muscle tissue samples. Similarly, statistical analysis of secondary outcomes of wound healing (e.g., neutrophilic infiltrate, fibroplasia, edema) also demonstrated statistically significant improvement in this same distribution. Although these markers do not directly prove clinical benefit, they are strongly suggestive.^15^

While there may be a trend suggesting that the protective hydrogel vehicle (HV) offers some benefit compared to the control, this study did not show a statistically significant improvement in bacterial reduction between the 2. This implies that the majority of the bacterial and wound healing benefits of HA were due to the 2 antibiotics contained in the product (i.e., vancomycin and tobramycin). To some extent, it is not surprising that these antibiotics produced the observed results. They are known to be active against the applied bacteria and wounds are known to heal better in the absence of pathogenic bacteria.^16–19^

## CONCLUSIONS

Wounds treated with HA had significantly lower bacterial presence compared to both the vehicle (HV) and control groups in skin and muscle tissue samples. Furthermore, the analysis of secondary wound healing outcomes showed a significant improvement in this distribution. In this porcine model of infected traumatic soft tissue injury, HA demonstrated significant benefits and further research and development to explore its use in a range of settings is justified.

### Limitations

There are several limitations to our study. Notably, the prespecified analysis of a cumulative logistic regression model did not converge. While we do believe this is due to our small sample size, it does limit the validity and generalizability of the study at this time. Additionally, our study was performed using a 7-day study endpoint. Although the primary outcome was successfully assessed, further studies may consider utilizing extended study timepoints to better elucidate long-term sequalae. Moreover, the blood samples drawn for detection of vancomycin and tobramycin at 1 h, and 1-, 3-, and 7 days after treatment were performed primarily as pilot testing of this assay. The exact absorption rates and amounts for both drugs from HA are unknown at this time and a dedicated study with multiple additional time points will be required to fully characterize product pharmacokinetics.

## Data Availability

The datasets generated during and/or analyzed during the current study are available from the corresponding author on reasonable request. The following medical technology is INVESTIGATIONAL and NOT AVAILABLE FOR COMMERCIAL SALE. This technology has NOT been approved by the FDA for human use.

## INSTITUTIONAL ANIMAL CARE AND USE COMMITTEE (IACUC)

Animal studies were in compliance with the applicable federal, state, and agency laws, policies, and guidelines for animal protection. This study was approved by Institutional Animal Care and Use Committee (IACUC) and U.S. Army’s Animal Care and Use Review Office (ACURO) approvals.

## INSTITUTIONAL CLEARANCE

Institutional clearance approved.
